# Regulation of Presynaptic Anchoring of the Scaffold Protein Bassoon by Phosphorylation-Dependent Interaction with 14-3-3 Adaptor Proteins

**DOI:** 10.1371/journal.pone.0058814

**Published:** 2013-03-14

**Authors:** Markus S. Schröder, Anne Stellmacher, Stefano Romorini, Claudia Marini, Carolina Montenegro-Venegas, Wilko D. Altrock, Eckart D. Gundelfinger, Anna Fejtova

**Affiliations:** 1 Department of Neurochemistry & Molecular Biology, Leibniz Institute for Neurobiology, Magdeburg, Germany; 2 Center for Behavioral Brain Science, Magdeburg, Germany; University of Iowa, United States of America

## Abstract

The proper organization of the presynaptic cytomatrix at the active zone is essential for reliable neurotransmitter release from neurons. Despite of the virtual stability of this tightly interconnected proteinaceous network it becomes increasingly clear that regulated dynamic changes of its composition play an important role in the processes of synaptic plasticity. Bassoon, a core component of the presynaptic cytomatrix, is a key player in structural organization and functional regulation of presynaptic release sites. It is one of the most highly phosphorylated synaptic proteins. Nevertheless, to date our knowledge about functions mediated by any one of the identified phosphorylation sites of Bassoon is sparse. In this study, we have identified an interaction of Bassoon with the small adaptor protein 14-3-3, which depends on phosphorylation of the 14-3-3 binding motif of Bassoon. In vitro phosphorylation assays indicate that phosphorylation of the critical Ser-2845 residue of Bassoon can be mediated by a member of the 90-kDa ribosomal S6 protein kinase family. Elimination of Ser-2845 from the 14-3-3 binding motif results in a significant decrease of Bassoon's molecular exchange rates at synapses of living rat neurons. We propose that the phosphorylation-induced 14-3-3 binding to Bassoon modulates its anchoring to the presynaptic cytomatrix. This regulation mechanism might participate in molecular and structural presynaptic remodeling during synaptic plasticity.

## Introduction

In brain synapses, the release of neurotransmitter is restricted to a specialized region of the presynaptic plasma membrane, the active zone, which is characterized by the presence of a dense proteinaceous matrix, called cytomatrix at the active zone (CAZ). The CAZ forms a distinct scaffold for multiple membrane trafficking processes occurring during neurotransmitter release [1]. One of the main constituents of the presynaptic CAZ is the large protein Bassoon [2]. Bassoon is necessary for proper organization of presynaptic release sites [3], efficient neurotransmitter release [4] and interference with Bassoon function leads to defects in short- and long-term plasticity [5], [6]. However, at the mechanistic level we do not understand the role of Bassoon and its modifications in these processes. Several independent proteomic studies identified Bassoon as one of the most heavily phosphorylated synaptic proteins [7], [8], [9]. Phosphorylation is a specific and reversible protein modification that can act as a molecular switch controlling protein function and it has been implied in the regulation of neurotransmitter release [10], [11]. However, to date functional consequences of Bassoon phosphorylation and how it does regulate its protein-protein interactions is not known.

Here, we have identified the small ubiquitous adaptor protein 14-3-3 as a novel interaction partner for Bassoon. The interaction critically depends on phosphorylation of the 14-3-3-binding motif of Bassoon. 14-3-3s are dimeric, highly abundant proteins with multiple cellular functions including regulation of signal transduction, cell survival and differentiation. They usually bind to phosphoserine-based motifs in their target proteins and often control the incorporation into multiprotein complexes and/or the subcellular localization of their binding partners [12]. Bassoon is anchored to the presynaptic CAZ and the underlying cytoskeleton by multiple protein-protein interactions with other CAZ constituents, including CAST/ELKS, Munc13, RIM, liprin-α and Piccolo/Aczonin, a paralogue of Bassoon [1], [2], [13]. Based on its tightly interconnected and highly organized nature the CAZ is considered as the principal scaffold spatially determining and organizing the sites of regulated neurotransmitter release at synapses. On the one hand, the CAZ seems to be a quite stable and tenacious structure with comparatively low molecular turnover of individual components [14], [15]. On the other hand, the CAZ is considered as a major substrate for presynaptic plasticity. Individual components display remarkable molecular dynamics [16], [17], [18], [19] and more recent reports suggest that various forms of synaptic plasticity are associated with profound molecular and structural remodeling of the CAZ on different time scales, i.e. from minutes to days [20], [21], [22]. Therefore, cellular mechanisms must exist that allow rearrangements of the CAZ, which should involve the dissociation of existing molecular interactions and the formation of new ones. In this study, we demonstrate that interference with the 14-3-3 binding to Bassoon results in a significant decrease of its molecular exchange rates at synapses of living neurons. We show that the specific phosphorylation on S2845 of Bassoon induces Bassoon-14-3-3 interaction and controls its dynamic association with the presynaptic cytomatrix. We propose that this regulation represents a common mechanism of inducing presynaptic molecular and structural remodeling during synaptic plasticity.

## Materials and Methods

### Antibodies

The following primary antibodies were used for Western blots: rabbit antibodies against pan 14-3-3 (α-pan 14-3-3; 1∶500; sc-629, Santa Cruz), 14-3-3η (α -14-3-3 η 1∶3,000; AB9736, Milipore-Chemicon), Bassoon sap7f (α-Bsn sap7f; 1∶2,000, [23]) and GFP (α-GFP, 1∶5,000; ab 6556; Abcam), mouse antibodies against Bassoon C-term (α-Bsn C-term; 1∶5,000; # 141 021 Synaptic Systems), Basoon m7f (α-Bsn m7f; 1∶1,000; Enzo Lifescience), GST (α-GST; 1∶10,000; Covance), His (α-His; 1∶1,000; Cell Signaling Inc.), Horseradish peroxidase conjugated antibodies against rabbit, mouse and guinea pig were purchased from Jackson ImmunoResearch Laboratories. Antibodies used for immunocytochemistry are: rat antibody against Homer (α-Homer; 1∶1000; AB5875; Milipore), guinea pig antibody against Synaptophysin (α-Synaptophysin; 1∶1000; 101004; Synaptic Systems), goat anti-rat and anti-guinea pig conjugated with Cy3 and Cy5 fluorophores, respectively (Invitrogen). Phosphorylation specific antibodies against Bassoon pS2845 (α-pS2845 Bsn) were derived from sera of rabbits immunized with KHL-coupled peptide CLQRSL-pS-DPK (containing amino acids 2840–2848 of rat Bassoon) by depletion against non-phosphorylated peptide and affinity purification using the phosphorylated peptide. (BioGenes GmbH). The antibodies were characterized for specificity against phosphoS2845 of Bassoon and used in a 1∶500 dilution for Western blot analyses.

### DNA constructs

The Bassoon fragments Bsn28 (aa 2715–3013) and GFP-Bsn (aa 95–3938) were described previously [24]. The mutations S2845A, S2845D and S2845E were introduced into Bsn28 by PCR using primers with mutated sequences and cloned into pBluescript-SKII+(Agilent Technologies). Wild-type and mutant Bsn28 fragments were cloned into pEGFP-C2 (Clontech Laboratories, Inc.) and pMito3-EGFP [25] vectors. GFP-Bsn^S2845A^ was generated by exchange of XmaJI/NdeI fragment containing S2845A (introduced by PCR using specific primers) in GFP-Bsn expression vector and the mutation was confirmed by sequencing. To generate His-Bsn11 (Bsn aa 2714–2867) the fragment was cloned via EcoRI/BamHI into the pET32a vector. 14-3-3η, 14-3-3β, 14-3-3γ and 14-3-3ε were generated using PCR on a rat brain Matchmaker cDNA library (Clontech Laboratories, Inc.) as template with extended primers to add EcoRI and XhoI restriction sites at the 5′ and 3′ ends of the fragments, respectively. The introduced restriction sites were used for cloning into pmRFP-C2 [25] or pGEX-5X1 (GE Healthcare) vectors.

### Yeast two-hybrid experiments

For a cDNA library screening, the Matchmaker Two-Hybrid System 3 (Clontech Laboratories, Inc.) was used with a pACT2 rat brain Matchmaker cDNA library (Clontech Laboratories, Inc.) as prey and Bassoon fragment Bsn28 (aa 2715–3013 of rat Bassoon) as bait. Transformation and selection was performed according to the manufacturer's protocols.

### Mitotargeting assay in COS-7 cells

Transfection of COS-7 cells (ATTC, Manassas, VA) grown on glass coverslips coated with Poly-D-Lysine (Sigma-Aldrich) was performed using the transfection reagent Polyfect (Qiagen) according to the manufacturer's protocol. After 24 h, cells were fixed in 4% paraformaldehyde and 4% sucrose in PBS, pH 7.4, for 5 min at room temperature and embedded in Mowiol 4–88. Images were taken with an upright microscope (Axioplan2; Zeiss) equipped with a camera (Cool Snap EZ camera; Visitron Systems) or Spot RT-KE; Diagnostic Instruments, Inc.) and MetaVue software (MDS Analytical Technologies). Constructs and procedure of the mitotargeting assay were described previously [25].

### Expression of proteins in HEK293T cells, pull downs and co-immunoprecipitations

HEK293T (ATTC, Manassas, VA) were grown in 6-well plates and transfected using the calcium phosphate method. In brief, 150 µl of 0.5 M CaCl_2_ were mixed with 4 µg DNA. Then, 50 µl of 140 mM NaCl, 50 mM HEPES, and 1.5 mM Na_2_PO_4_, pH 7.05, were added and, after 1 min, applied to cells in culture. The cells were incubated for 4 to 6 h at 37°C in 5% CO_2_ atmosphere before exchanging growth media. Cells were lysed in 10 mM HEPES, pH 7.5, 150 mM NaCl, 0.5% Triton-X-100, for pull downs, or 10 mM Tris-HCl, pH 8.0, 150 mM NaCl, 1% NP-40, 10% glycerol, for co-immunopreciptations, containing Complete protease inhibitors (Roche) and PhosStop (Roche) for 10 min at 4°C. Insoluble material was removed by centrifugation. For dephosphorylation of proteins 10 units of calf intestine alkaline phosphatase (Fermentas) were added to the cell lysates and incubated for 1 h at 37°C. For pull downs 25 µg purified GST or 50 µg purified GST-14-3-3h were coupled to glutathion sepharose Fast Flow (Amersham) and incubated with cell lysates overnight at 4°C. After washing with lysis buffer bound material was eluted by incubation with 10 mM glutathione, 50 mM Tris-HCl, pH 7.5, for 30 min at RT. Co-immunoprecipitations were done using MicroMACS anti-GFP MicroBeads and MicroColumns (Miltenyi Biotec) according to the manufacturer's protocol but using the lysis buffer in all washing steps

### Mouse strains and brain extract preparation

Bassoon mutant mice (BsnΔEx4/5; [4]) were used for blot-overlay experiments and for characterization of α-pS2845 Bsn. Mice were killed by neck dislocation and brains were removed and homogenized in 320 mM sucrose, 25 mM Tris-HCl, pH 7.4, containing Complete protease inhibitor and PhosStop, and centrifuged at 1,000×g for 10 min. The supernatant was centrifuged at 12,000×g for 20 min and the pellet was resuspended in 25 mM Tris-HCl, pH 7.4, 150 mM NaCl, 1% Triton-X-100 to obtain the crude membrane fraction (pellet P2). All steps were performed at 4°C. For hyperphosphorylation of proteins 5 mM MgCl_2_ and 1 mM ATP (Sigma-Aldrich) was added and incubated at 30°C for 30 min. The reaction was stopped by addition of 10 mM EDTA. Protein concentrations were determined by amidoblack assay, the samples were adjusted by adding of 1×loading buffer to equal protein concentrations and loaded on SDS polyacrylamide gels for subsequent Western blotting. All experiments involving animals were carried out in accordance with the European Committees Council Directive (86/609/EEC) and approved by the local animal care committee (Landesverwaltungsamt Sachsen-Anhalt, AZ: 42502/2-988 IfN).

### Western blot and blot overlay

Western blots for the detection of Bassoon were performed using 3.5–8% Tris-acetate gels and transferred to PVDF membranes by tank blotting [4]. For the detection of 14-3-3 proteins 5–20% Tris-glycine gels were used. For immunodetection, blots were blocked with 1% BSA in PBS with 0,1% Tween-20, incubated with primary antibodies for 1 h at room temperature and after several washing steps probed with peroxidase coupled secondary antibodies for 1 h at room temperature. The visualization was performed by chemiluminescent detection (Pierce or Millipore) and detected with Amersham hyperfilms (GE Healthcare) or a ChemoCam Imager HR16-3200 (Intas Science Imaging). For blot-overlay experiments each blot was incubated with 25 µg of purified GST or 50 µg GST-14-3-3η fusion proteins for 1 h at RT and subsequently processed for immunodetection of the bound proteins.

### Neuronal cultures, immunostainings

Dissociated primary hippocampal and cortical cultures were prepared [26] and transfected with a calcium phosphate method [24] as described previously. Neurons were generally transfected at 3–4 day in vitro (DIV) and analyzed at 14–16 DIV. The transfected cells were fixed and images were acquired as it was described above for COS7 cells. The analysis of co-localization of proteins expressed in neurons was performed using the ‘Colocalization Analysis’ plug-in of image J [27], [28]. To analyze of synaptic targeting of GFP-Bsn and GFP-Bsn^S2845A^, transfected neurons were fixed and stained for synaptic markers Homer and Synaptophysin. Bsn-positive puncta were detected using Openview [19]. Homer and Synaptophysin intensities were then measured at the location of Bsn-positive puncta in the according channels. After subtraction of an intensity treshhold to exclude background levels the percentage of Bsn-positive puncta also positive for Homer or Synaptophysin was calculated.

### In vitro phosphorylation of Bassoon

His-Bsn11 and the His tag alone was expressed in bacteria (BL21 Codon Plus (DE3) RIPL; Stratagene) and purified following the manufacturer's protocol. 5 µmol of His-fusion proteins were incubated with 0 to 40 mU 90-kDa ribosomal S6 protein kinase (RSK) protein (RSK1 #14-509, RSK2 #14-408, RSK3 #14-462, RSK4 #14-702; Millipore) in 50 mM Tris-HCl, pH 7.5, 10 mM MgCl_2_, 1 mM EGTA, 1 mM DTT, 1 mM ATP for min at 37°C. Phosphorylation of proteins was analyzed by Western blots using phospho-specific antibodies.

### Live imaging microscopy, fluorescence recovery after photobleaching (FRAP) experiments

Live imaging was performed at 37°C using an inverted microscope (Observer. D1; Zeiss) in a heated imaging chamber TC-344B (Warner Instruments) and an EMCCD camera (Evolve 512; Photometrics) controlled by MetaMorph Imaging (MDS Analytical Technologies) and VisiView (Visitron Systems GmbH) software. The FRAP laser DL-473 (Rapp Optoelectronics) was driven by the FRAP targeting device Visifrap 2D (Visitron Systems GmbH). Videos were taken prior to bleaching 10 s (10 pictures, 250 ms exposure time), then for fluorescence bleaching a laser pulse of ms was applied and the recovery was monitored for 25 s (100 pictures, 250 ms exposure) and additional 300 s (300 pictures, 250 ms exposure). For image analyses ImageJ [28] and MetaMorph Imaging (MDS Analytical Technologies) were used. Recovery rate was determined after background subtraction (from three independent spots) and bleaching correction, which was performed by selection of five fluorescence spots which were not used for FRAP, by calculating the ratio of the spot intensity for every time point versus the intensity before (set to 100%) and after (set to 0%) bleaching.

For vesicle mobility experiments video streams of 25-s recorded at 4 Hz with an exposure time of 250 ms were analyzed using MetaMorph software. To determine velocities and running distances of vesicles carrying GFP-Bsn or GFP-Bsn14-3-3BM, traces of mobile particles were visualized on kymographs of axonal segments. Traces showing processive movement (without stops and changes in velocities or movement directions) were analyzed. Statistical analysis of all data was done with the software GraphPad Prism 5 (GraphPad Software).

## Results

### Bassoon interacts directly with 14-3-3 proteins in a phosphorylation-dependent manner

**Figure 1:**
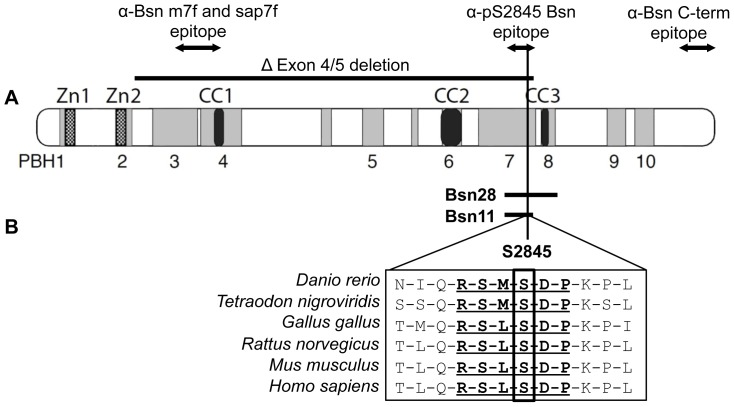
Bassoon contains a 14-3-3 interaction interface. (A) Domain structure of Bassoon with the position of the fragments Bsn28 and Bsn11 (horizontal bars) containing serine-2845 (vertical line) critical for 14-3-3η interaction are depicted. (PBH 1-10: Bassoon/ Piccolo homology domains 1-10; Zn: Zinc finger domain; cc: coiled coil domain) The deletion of exon 4 and 5 in BsnΔEx4/5 mutant mice and positions of epitopes of antibodies used in the study are depicted. (**B**) Alignment of the amino acid sequences of the region containing the 14-3-3 binding motif from Bassoon orthologues of different species is shown. The RSM/LpSDP 14-3-3 binding motif is bold and the critical serine residue S2845 is boxed.

**Figure 2:**
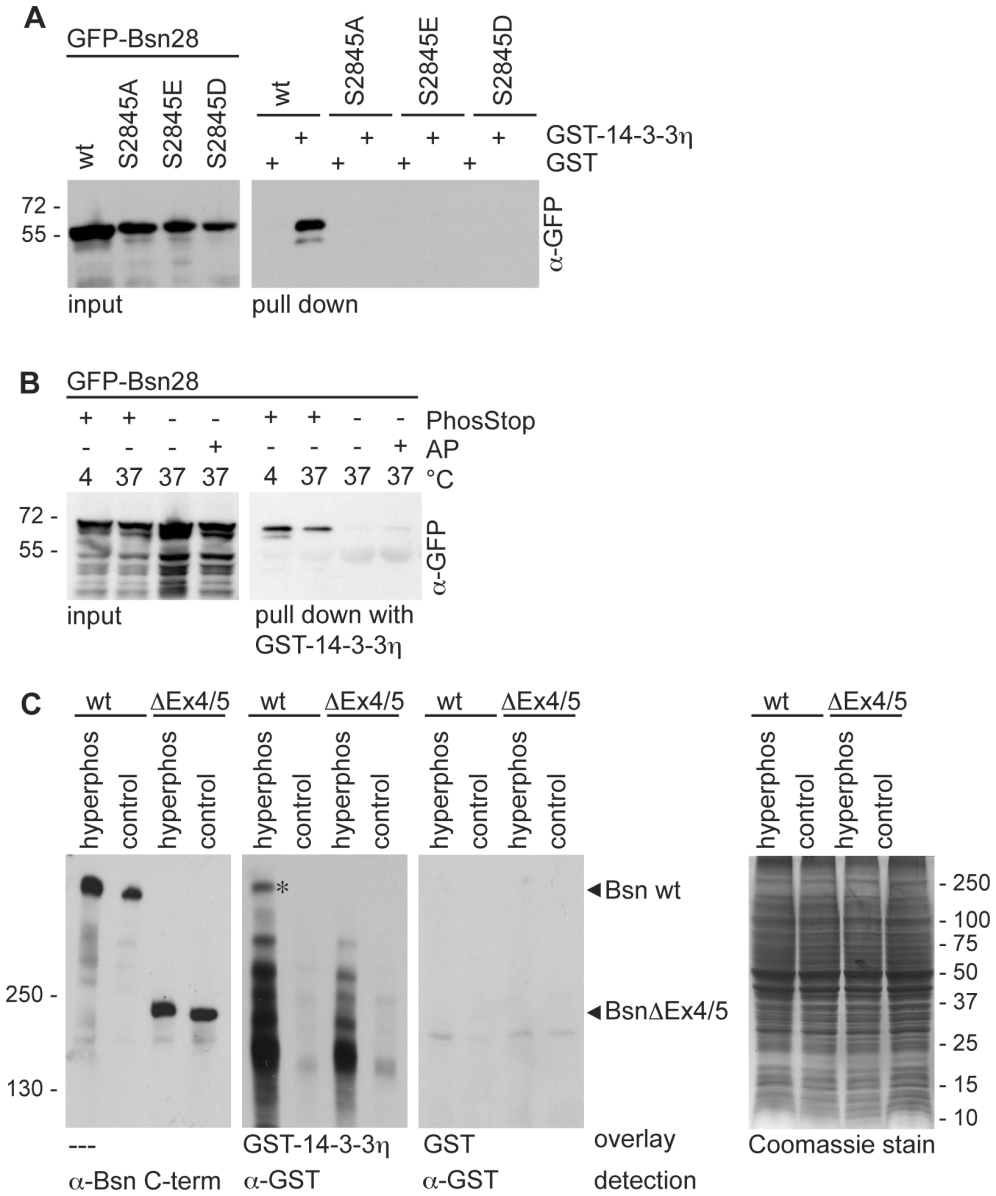
Direct binding of Bassoon to 14-3-3 depends on phosphorylation of Serine-2845. (A) GFP-Bsn28 wild-type (wt) and its variants containing mutation in the critical S2845 residue (S2845A, S2845E, S2845D) were expressed in HEK293T cells and employed for pull-down experiments with bacterially expressed and purified GST-14-3-3η or GST as a control. Detection of GFP-tagged proteins in the cell lysates (input) and the bound fractions (pull down) was performed using α–GFP antibody. GFP-Bsn28 was successfully co-precipitated by GST-14-3-3η, but not with GST. All tested mutations interfered with the binding. **(B)** Cell lysates from HEK293T cells expressing GFP-Bsn28 without any additives or supplemented with phosphatase inhibitors (PhosStop) or alkaline phosphatase (AP) and incubated at 4° or 37° C were used for pull-down experiments with immobilized GST-14-3-3η. GFP-Bsn28 was detected in cell lysates (input) and the bound fractions (pull down) using α-GFP antibodies. **(C)** Hyperphosphorylated and control P2 fractions from brains of wild-type (wt) and Bassoon mutant mice (BsnΔEx4/5) were separated by SDS-PAGE. Equal amounts of protein in each sample was controlled by Coomassie Blue staining for all proteins (right panel). Immunodetection with α-Bsn C-term antibodies revealed the immunoreactivity of wild-type Bassoon (420 kD) and the mutant BassoonΔEx4/5 residual protein (180 kD). Purified GST-14-3-3η or GST fusion proteins were used for the overlay and detected by α-GST antibody. Note the presence of the band (marked by asterisk) corresponding to Bassoon in lysates from wt but not from BsnΔEx4/5 mice showing binding GST-14-3-3h, but not GST fusion protein. Bars and number on the left side of the blots and on the right side of Coomassie-stained gel show sizes and positions of molecular weight markers. Images shown in this panel are representative for results obtained in at least two independent experiments

Initial clues for an interaction of Bassoon with 14-3-3 proteins derived from a yeast-two-hybrid (Y2H) screen of a rat brain cDNA library with the cDNA fragment Bsn28 covering amino acid residues 2715 – 3013 of rat Bassoon as a bait (Fig. 1A). One interacting clone contained the full coding sequence of 14-3-3η. Consensus 14-3-3-interaction motifs were defined previously [29], [30], [31] and could be identified using online accessible tools such as The Eukaryotic Linear Motif resource [32]. Amino acid residues 2842–2847 of rat Bassoon with the sequence RSLSDP form a putative interaction site for 14-3-3, which fits to the described classical mode-1 binding motif (RSXpSXP) [29]. This sequence is highly conserved between all analyzed Bassoon orthologues (Fig. 1B) suggesting a high evolutionary pressure for the integrity of the 14-3-3 binding interface on Bassoon. To confirm that this particular motif mediates an interaction of Bassoon with 14-3-3η a series of pull down experiments was performed. To this end GST-tagged 14-3-3η was expressed and affinity purified from bacteria. The GFP-tagged Bassoon fragment Bsn28 (GFP-Bsn28) and the corresponding mutant (GFP-Bsn28^S2845A^), in which the critical serine residue S2845 was changed to alanine, were expressed in HEK 293T cells. The expression of the Bassoon fragments in mammalian cells was chosen to allow the phosphorylation of the binding motif, which was previously reported to be a necessary prerequisite for 14-3-3 binding to almost all reported targets [33]. Purified GST-14-3-3η, but not GST alone, successfully pulled down GFP-Bsn28 from the cell lysates. The mutated Bassoon fusion protein GFP-Bsn28^S2845A^ showed no binding to GST-14-3-3h confirming that the intact S2845-containing motif was required for interaction of Bassoon with 14-3-3η (Fig. 2A). To test whether phosphorylation of S2845 is required for the interaction with 14-3-3η, cell lysates of HEK293T cells expressing GFP-Bsn28 were prepared without the addition of phosphatase inhibitors to destabilize the phosphorylated state of the expressed proteins or the cell lysates were treated with alkaline phosphatase to dephosphorylate them prior to the pull down experiment. In both conditions the pull-down efficiency was strongly diminished compared to the controls containing phosphatase inhibitors. Accordingly we conclude that the interaction of 14-3-3 critically relies on the phosphorylation of S2845 of Bassoon, which is in line with the phosphorylation dependency of 14-3-3 protein interactions (Fig. 2B). To mimic the phosphorylation of S2845 we substituted this residue by glutamate or aspartate. However, none of the phosphomimetic mutants was able to bind 14-3-3η (Fig. 2A), which is in agreement with the previously described high selectivity of 14-3-3 to phosphoserine- or phosphothreonine-containing binding motifs [34].

In order to address the question, whether the physical interaction of Bassoon and 14-3-3 proteins is direct we performed a blot-overlay assay. For this goal, control and hyperphosphorylated brain extracts of adult wild-type (wt) and Bassoon mutant mice (BsnΔEx4/5; [4]) were separated by SDS-PAGE and transferred to PVDF membrane. In BsnΔEx4/5 mice the exons 4 and 5 of Bassoon coding for the amino acids 505 to 2889 were deleted. Accordingly, these mice lack the identified 14-3-3 binding site around S2845, in the residual Bassoon fragment. The immunodetection with specific anti-Bassoon antibodies (α-Bsn C-term) revealed the expected bands of 420 kDa in the wild-type and of 180 kDa in the BsnΔEx4/5 extracts (Fig. 2C). The protein hyperphosphorylation driven by endogenous protein kinases achieved by incubation of brain lysates in the presence of 5 mM MgCl_2_ and 1 mM ATP at 30°C for 30 min leads to a shift of the Bassoon bands to higher molecular weights suggesting a change in the charge and conformation of Bassoon due to increased phosphorylation. Further blot membranes prepared in parallel were incubated with purified recombinant GST or a GST-14-3-3η fusion protein. Immunodetection with α-GST antibodies showed the binding of GST-14-3-3η at the exact location of the wild-type Bassoon band in lysates from wild type animals but not in lysates from BsnΔEx4/5 mice indicating the interaction of 14-3-3 with Bassoon (Fig. 2C). The interaction was specific for 14-3-3h since GST alone did not bind to proteins immobilized on the blot membrane. Due to the denaturing conditions and the separation of the brain extracts by SDS-PAGE the binding could not involve any other proteins and was therefore direct. The overlay experiment was successful only when hyperphosphorylated brain extracts were used suggesting a low abundance and/or a short lifetime of pS2845 Bassoon in adult mouse brain.

### 14-3-3 interacts with Bassoon in living mammalian cells

**Figure 3:**
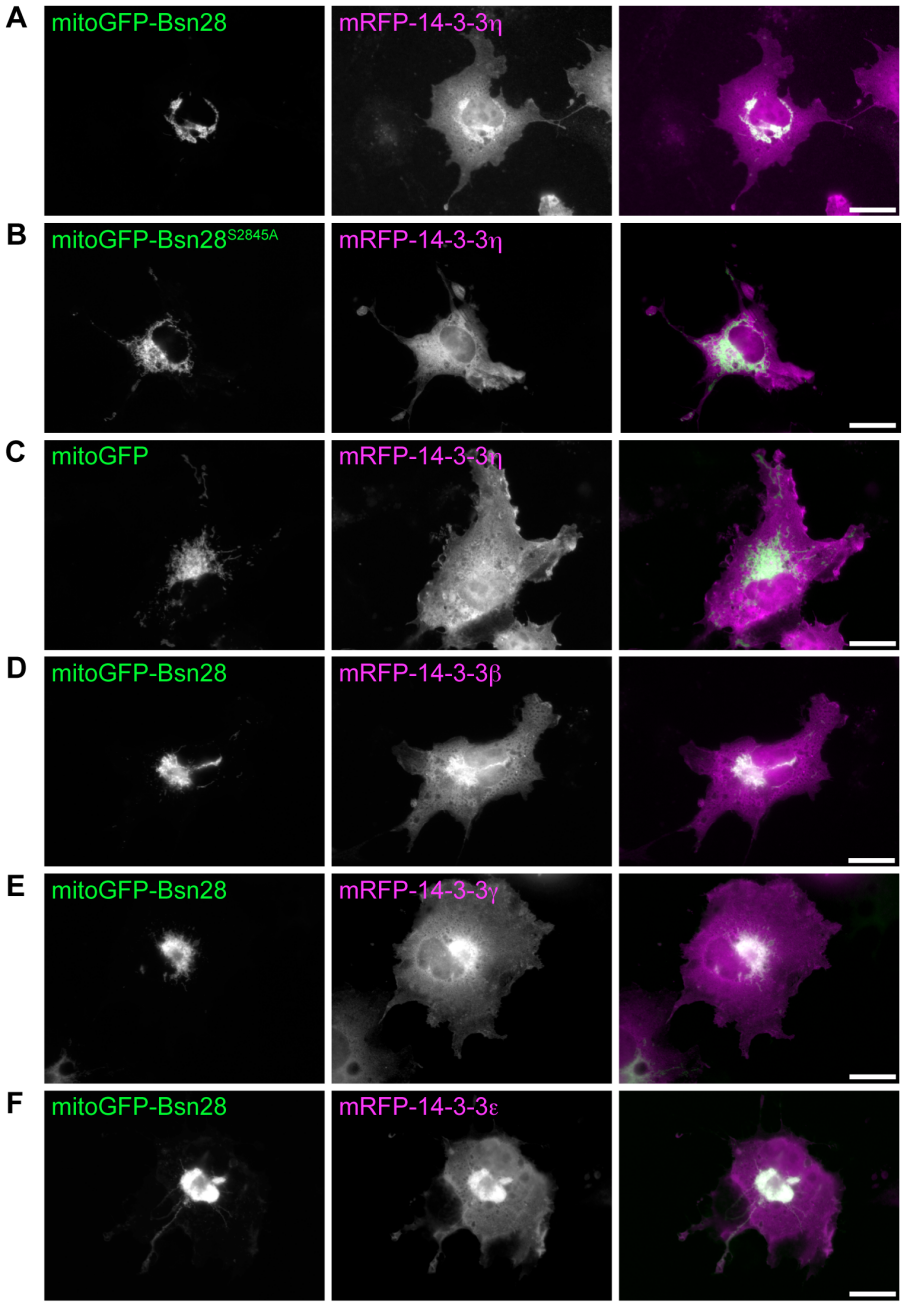
The 14-3-3-interaction motif of Bassoon can recruit 14-3-3 proteins in living cells. MitoGFP-Bsn28 **(A, D-F)**, its mutant GFP-Bsn28^S2845A^ **(B)** and mitoGFP **(C)** expressed in COS7 cells are targeted to mitochondria. mRFP-14-3-3η shows a diffuse cytoplasmic localization when coexpressed with mitoGFP-Bsn28^S2845A^ **(B)** or mitoGFP **(C)** but is strongly enriched at mitochondria in cells expressing mitoGFP-Bsn28 **(A)**. mRFP-tagged β, γ and ε isoforms of 14-3-3 are also recruited to mitochondria expressing mitoGFP-Bsn28 **(D-E)**. Representative images from one of three **(A-C)** or two **(D-E)** independent experiments are shown. Scale bars are 20 µm.

**Figure 4:**
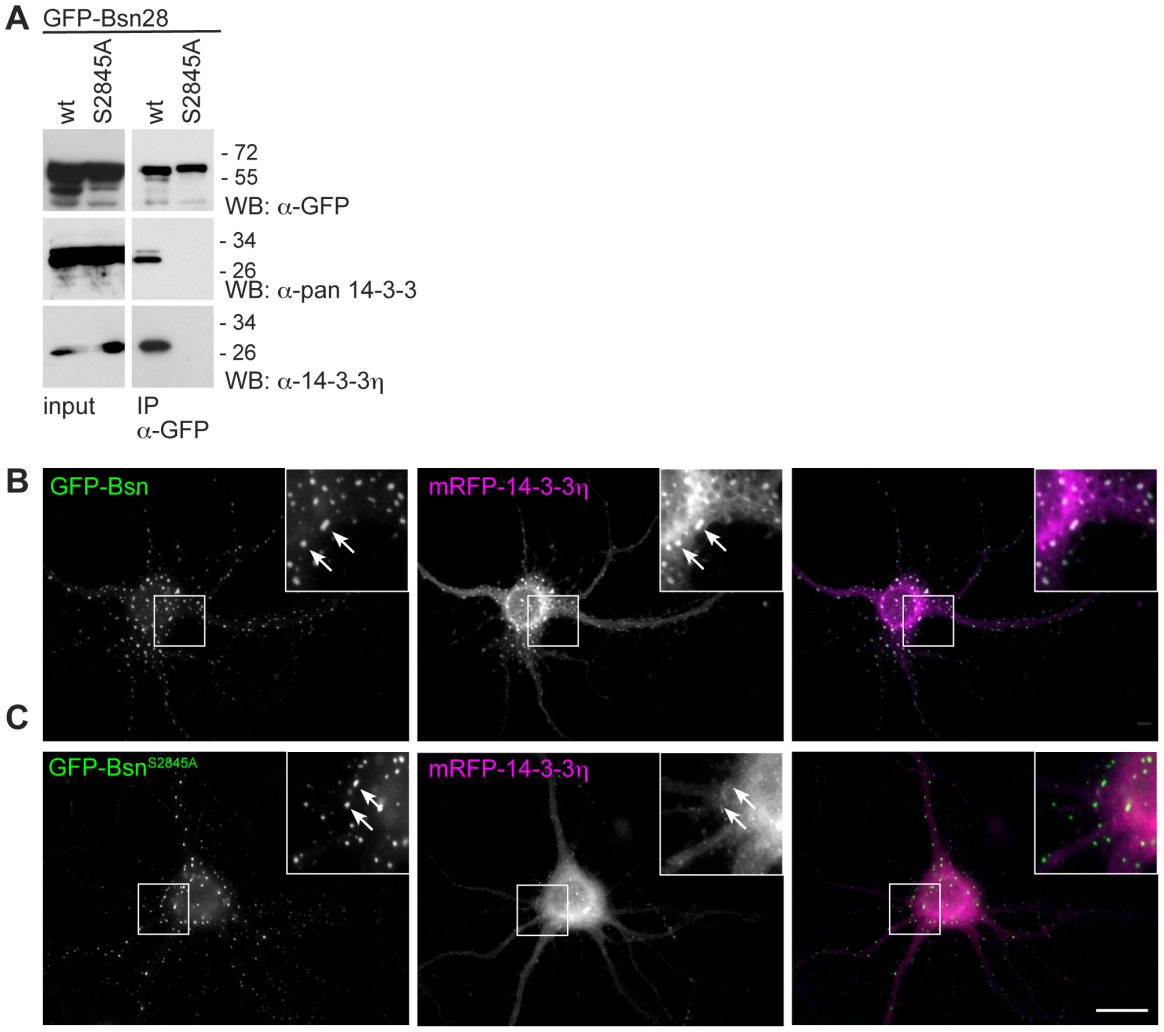
Bassoon interacts with 14-3-3 in living cells. (A) GFP-Bsn28 (wt) wild-type or its mutant GFP-Bsn28^S2845A^ were expressed in HEK293T cells and cell lysates were used for immunoprecipitations with α-GFP antibody. Successful expression of GFP-tagged proteins was shown in cell lysates (input) and bound fraction (IP) using a-GFP antibody. The endogenously expressed 14-3-3 proteins were co-precipitated with GFP-Bsn28 but not with its mutant Bsn28^S2845A^ as demonstrated by detection with α-pan 14-3-3 and α-14-3-3η antibody. Note two bands detected in the bound fractions using α-pan 14-3-3 antibody suggesting immunoprecipitation of multiple 14-3-3 isoforms. The bars and number on left side of blots show the sizes and positions of molecular weight markers. Images shown are representative for results obtained in two independent experiments. **(B, C)** mRFP-14-3-3η is localized to clusters formed by GFP-Bsn (arrows in **B**) but not to clusters formed by the corresponding 14-3-3 binding mutant GFP-Bsn^S2845A^ (arrows in **C**) in transfected primary hippocampal neurons. Scale bar is 20 µm.

To support our interaction data we performed the previously described mito-targeting assay [25]. The wild-type and the mutated fragments of Bassoon, Bsn28 and Bsn28^S2845A^, which were successfully used in the pull-down experiments, were fused to a mitochondrial targeting sequence leading to their anchoring to the outer membrane of mitochondria upon expression in COS7 cells. The co-expression of the mitochondria-targeted Bassoon fragment (mitoGFP-Bsn28) and an mRFP fusion protein of 14-3-3η (mRFP-14-3-3η) results in a clear enrichment of mRFP-14-3-3η at mitochondria carrying mitoGFP-Bsn28 (Fig. 3A). When expressed together with the mutated Bassoon fragment (mitoGFP-Bsn28^S2845A^) or with mitochondria-targeted GFP (mitoGFP), mRFP-14-3-3η showed a diffuse cellular localization (Fig. 3B, C). This demonstrates that Bassoon can interact with 14-3-3 in living mammalian cells and that this interaction depends on the intact 14-3-3 binding motif containing S2845 of Bassoon. The 14-3-3 isoforms mRFP-14-3-3β, γ and ε did also interact with Bassoon in this assay (Fig. 3D-F). Furthermore, we were able to co-immunoprecipitate endogenous 14-3-3 proteins with heterologously expressed GFP-Bsn28, but not with GFP-Bsn28^S2845A^ in HEK293T cells (Fig. 4A), showing the ability of endogenous 14-3-3 to bind to the respective binding interface of Bassoon. While the detection with a-14-3-3η antibodies showed the co-immunoprecipitation of the η isoform the α-pan 14-3-3 antibodies detected two distinct bands. The less prominent upper band represents the 14-3-3ε isoform, while the other isoforms run at the height of the more prominent lower band [35]. Both experiments confirmed the critical importance of S2845 of Bassoon for its interaction with different 14-3-3 isoforms.

Next we investigated, whether Bassoon interacts with 14-3-3η in neurons. To this end primary hippocampal neurons were co-transfected with mRFP-14-3-3η and a GFP-tagged construct comprising the amino acids 95 to 3938 of rat Bassoon (GFP-Bsn). This construct behaves in many respects like wild-type Bassoon when expressed in primary neurons [14], [24], [36], [37]. Additionally, we created a 14-3-3 binding-deficient Bassoon construct (GFP-Bsn^S2845A^) by introducing the point mutation S2845A, which disrupted the binding of Bassoon to 14-3-3 proteins in vitro, into GFP-Bsn. In accordance with previous observations, both GFP-fusion proteins were localized at synapses in a similar way as the endogenous Bassoon protein. In addition, GFP-Bsn was localized in ectopic cytoplasmic clusters formed by mis-targeted over-expressed protein in the transfected neurons [24], [25]. Co-transfected mRFP-14-3-3η could be found at synapses when co-expressed with both GFP-Bsn and its S2845A mutant, which is likely due to interaction with its multiple synaptic binding partners. Interestingly, mRFP-14-3-3η was recruited to ectopic cytoplasmic clusters (Fig 4B) only in cells expressing GFP-Bsn but showed a diffuse cytoplasmic localization when co-expressed with GFP-Bsn^S2845A^ (Fig. 4C). We analyzed the degree of co-recruitment by calculation of Pearsons correlation coefficient [27] for mRFP and GFP fluorescence in double transfected cells. This coefficient was significantly higher for mRFP-14-3-3η expressed with GFP-Bsn than for GFP-Bsn^S2845A^ (0,66±0,03 vs. 0,54±0,03 mean±SEM, n = 15 vs. 12 cells, P<0.01, unpaired t-test). This demonstrates that Bassoon can sequester 14-3-3η in neurons and that this is crucially dependent on presence of S2845 residue in Bassoon. Since the GFP-Bsn^S2845A^ construct did not recruit 14-3-3η we assume that the described binding motif represents the only interaction interface for 14-3-3 in Bassoon.

### Generation of a phosphorylation specific antibody for pS2845 of Bassoon

**Figure 5:**
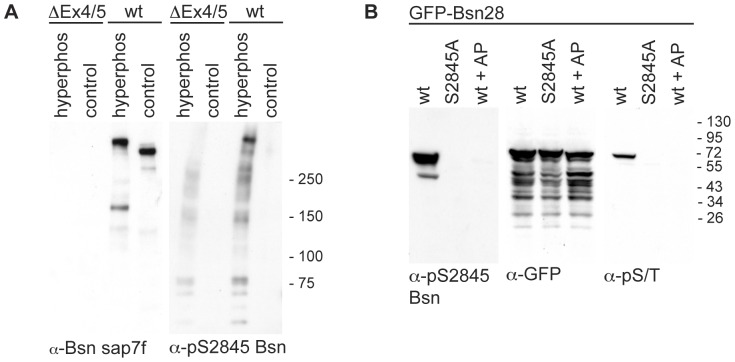
Characterization of the antibody against phosphorylated S2845 of Bassoon. **(A)** α-pS2845 Bsn antibody was tested on P2 fractions from brains of wild-type (wt) and Bassoon mutant (BsnΔEx4/5) mice. Bassoon was detected by α-Bsn sap7f antibody in the samples from wt mice but not from mutant. The Western blot, which was prepared in parallel and incubated with the phosphorylation-specific antibody α-pS2845 Bsn preferentially detects Bassoon in the hyperphosphorylated sample and showed only weak unspecific immunoreactivity in the samples of the Bassoon-mutant mice. Images shown are representative for independent experiments done with lysates obtained from three pairs of mice. **(B)** GFP-Bsn28 (wt) or its mutant (S2845A) were expressed in HEK293T cells. The cell lysates were either treated with phosphatase inhibitors to prevent dephosphorylation or the alkaline phosphatase (AP) was added to reduce phosphorylation of the proteins. Comparable expression of all constructs was demonstrated by the α-GFP staining. Immunodetection using α-pS/T antibodies revealed that GFP-Bsn28 but not S2845A mutant is phosphorylated under the tested conditions. α-pS2845 Bsn recognized only the phosphorylated GFP-Bsn28 but not S2845A mutant and the dephosphorylated GFP-Bsn28 in samples treated with AP. Images shown are representative for one of at least three independent experiments. The bars and number on left side of blots show the sizes and positions of molecular weight markers.

In order to analyze the phosphorylation state of S2845 of Bassoon, which is critical for its interaction with 14-3-3, we generated a phosphorylation specific antibody (further on named α-pS2845 Bsn). To test the specificity of this antibody for Bassoon, we tested it on brain extracts (membrane enriched P2 fractions) from wild-type and BsnΔEx4/5-mutant mice. The α-pS2845 Bsn antibody reliably recognized a band with a molecular weight of ∼450 kDa corresponding to Bassoon in hyperphosphorylated extracts from wild-type mice. No corresponding immunoreactivity was observed in the extracts from mutant mice, confirming the specificity of this antibody for Bassoon (Fig. 5A). To test the specificity of the antibody for phosphorylated S2845 we tested it on lysates from HEK293T cells over-expressing GFP-Bsn28 or its mutant GFP-Bsn28^S2845A^. The α-pS2845 Bsn antibody recognized GFP-Bsn28 but failed to recognize its S2845A mutant or dephosphorylated GFP-Bsn28 in cell lysates treated with alkaline phosphatase (Fig. 5B). The effective dephosphorylation of GFP-Bsn28 was confirmed by immunodetection with an antibody specific to phosphorylated serine and threonine (α-pS/T). These experiments demonstrated that the α-pS2845 Bsn antibody does not recognize non-phosphorylated S2845 of Bassoon.

### The 14-3-3 binding site on Bassoon can be phosphorylated by RSK-family protein kinases

**Figure 6:**
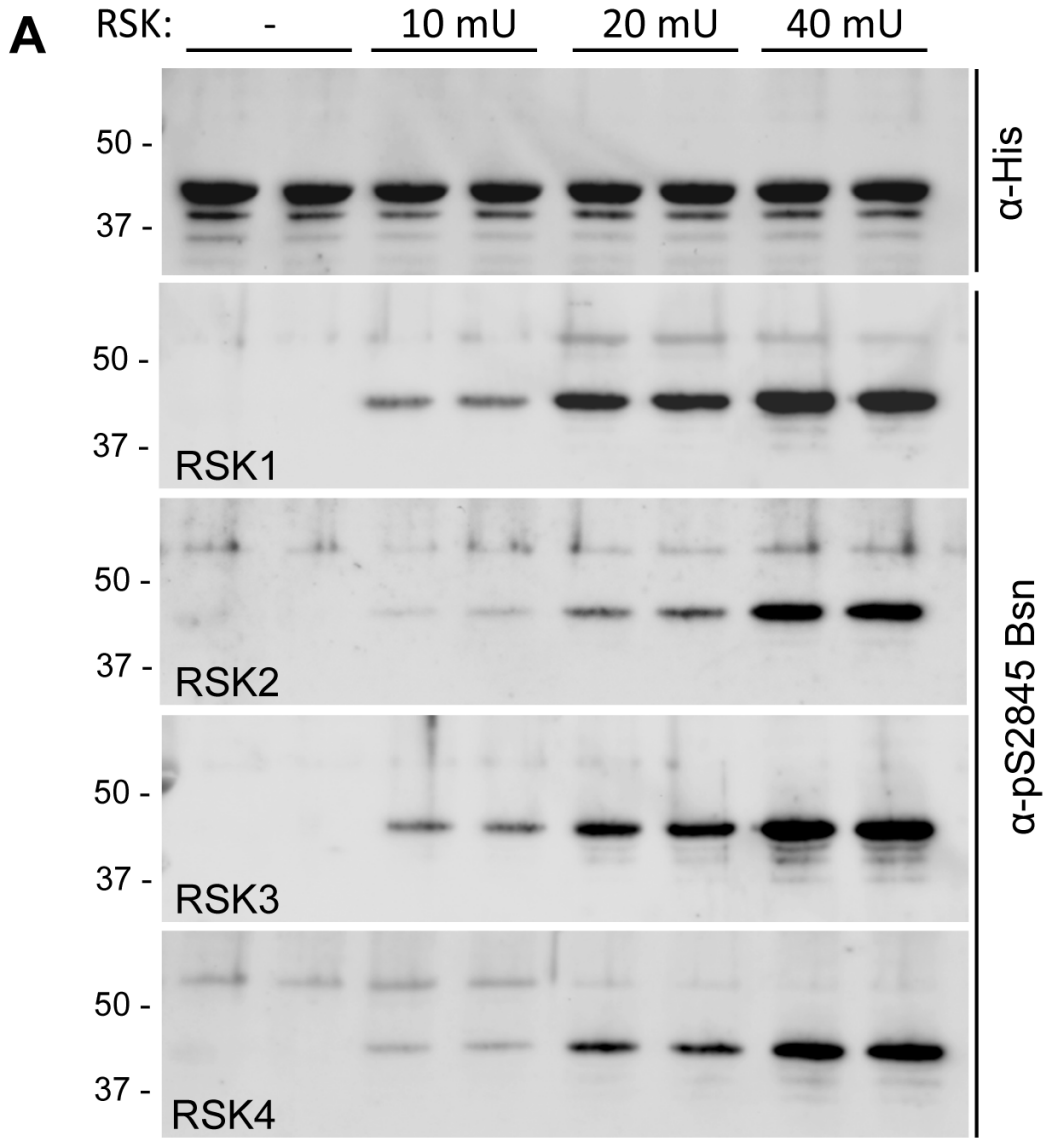
Protein kinases of the RSK family phosphorylate Bassoon S2845 in vitro. Bacterially expressed, affinity-purified His-Bsn11 was phosphorylated in vitro by RSK 1, 2, 3 and 4 using ascending amounts (10-40 mU) of active purified kinase. Equal loading of His-Bsn11 in the assay was controlled by immunodetection using α-His in all samples. Phosphorylation of His-Bsn11 was monitored by immunodetection with α-pS2845 Bsn, which revealed increasing phosphorylation depending on the increasing concentrations of kinases. The images shown here are representative for three independently performed experiments. The bars and numbers on the left side of the blots show the sizes and positions of the molecular markers.

To understand the physiological context under which the phosphorylation of Bassoon on S2845 occurs, it is important to identify the protein kinases driving this modification. To address this question we used in silico predictions of the online services NetPhosK [38] and MnM 3.0 [39], which both pointed towards a possible phosphorylation by protein kinases of the 90 K ribosomal protein S6 kinase (RSK) family. To test this prediction we performed in vitro phosphorylation of a bacterially expressed, affinity-purified His-tagged fusion protein of the Bassoon fragment Bsn11 (His-Bsn11, amino acid residues 2714-2867, Fig. 1), which includes the 14-3-3 binding interface, with commercially available activated protein kinases RSK1, 2, 3 and 4 (Fig. 6). Successful phosphorylation of Bassoon S2845 was monitored by immunodetection with the α-pS2845 Bsn antibody. The degree of S2845 phosphorylation increased with increasing amounts of added kinases, confirming the specificity of the phosphorylation reaction. All kinases tested directly phosphorylated the Bassoon fragment even at the lowest tested concentration. However, the RSK1 and RSK3 were more efficient than RSK2 and RSK4. Thus, the members of RSK family are good candidates to control the molecular switch regulating the interaction of Bassoon with 14-3-3.

### Mutation of the 14-3-3 binding site influences molecular dynamics of synaptic Bassoon

**Figure 7:**
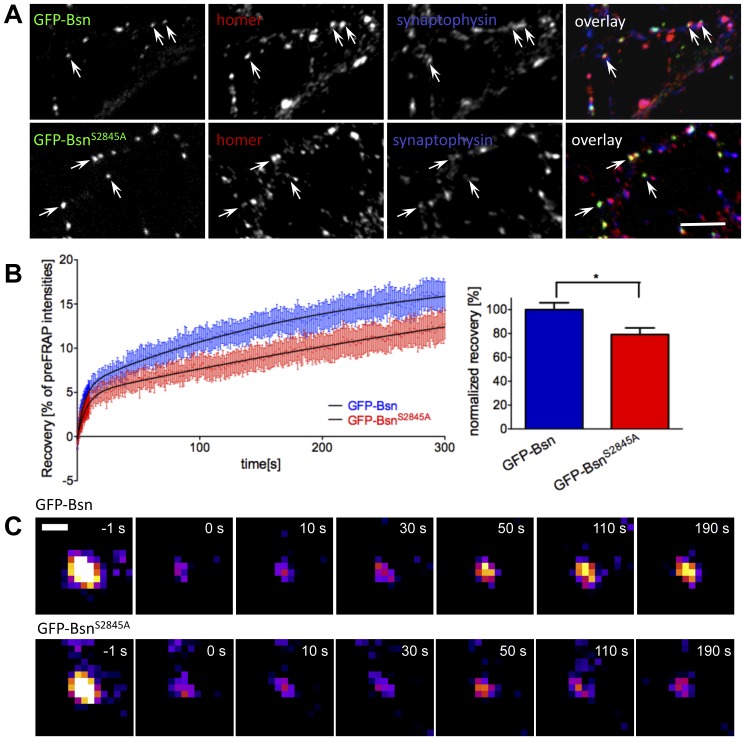
Analysis of molecular dynamics of GFP-Bsn and GFP-BsnS2845A by FRAP. (A) Primary hippocampal neurons were transfected with GFP-Bsn or GFP-Bsn^S2845A^ at DIV3 and analyzed at DIV 14-16. Synaptic targeting of both constructs was assessed by co-staining with synaptic markers homer and synaptophysin. The arrows highlight synapses of transfected cells stained for both markers. Scale bar, 20 µm. **(B)** Curves in the left panel show averaged fluorescence recovery of all analyzed puncta plotted as actual spot intensity relative to pre-bleaching intensity. The whiskers show SEM for each value. GFP-Bsn (wild-type) shows a higher recovery rate compared to GFP-Bsn^S2845A^. Recovery was significantly lowered for GFP-Bsn^S2845A^ 300 s after photobleaching. Columns in right plot represent mean value normalized to GFP-Bsn recovery, whiskers SEM, * indicates P<0.05. The values were obtained from 4 independent imaging sessions. **(C)** Representative example image showing bleaching and recovery of a GFP-Bsn and GFP-Bsn^S2845A^ puncta. Scale bar is 1 µm.

In many cases 14-3-3 induces a spatial redistribution of its binding partners [33]. Therefore we investigated whether the 14-3-3 binding to Bassoon influences its synaptic localization. To this end we expressed GFP-Bsn and its 14-3-3 binding mutant (GFP-Bsn^S2845A^) in cultured hippocampal neurons. Both constructs showed an identical general cellular distribution and degree of co-localization with synaptic markers (Fig. 7A; GFP-Bsn vs. GFP-Bsn^S2845A^: 89±4 vs. 93±2% co-localization with synaptophysin, and 94±2 vs. 92±2% with homer, mean±SEM, n = 6 images for each quantification, P>0.05 in one way ANOVA with Bonferoni posttest). To check, whether the 14-3-3 interaction influences the dynamic properties of synaptic Bassoon we applied a FRAP assay (Fig. 7A). The long half-life and the tight anchoring of Bassoon to the presynaptic active zone lead to low recovery rates (16% of initial intensity within 5 min after bleaching for GFP-Bsn), which is in agreement with previously published data [14]. Our analyses have shown that the mutation of the 14-3-3-binding site even further decreased the Bassoon recovery (Fig. 7 B,C; GFP-Bsn vs. GFP-Bsn^S2845A^: 100±6 vs. 79±6%, mean±SEM, normalized to recovery of GFP-Bsn, n = 43 vs. 30 videos, P = 0.0136, unpaired t-test). The quantitative analysis of the fluorescence intensities of GFP-Bsn^S2845A^ puncta showed no significant difference compared to GFP-Bsn puncta (100±19 vs. 101±18% mean±SEM, normalized to intensity of GFP-Bsn, n = 42 vs. 30 videos, P = 0.75, unpaired t-test) indicating that the decreased recovery cannot be explained by differences in the expression levels of both constructs.

Bassoon and its paralogue Piccolo are transported into the axon on membranous organelles [25], [40], [41], [42]. To check whether the defect in supply of mobile GFP-Bsn^S2845A^ contributes to observed decreased recovery we analyzed the mobility properties of fluorescently-labeled vesicles in neurons transfected with wt and mutant constructs. We measured the vesicle velocities, the travelled distances of the vesicles and the amount of moving vesicles in relation to stable puncta. The velocity of vesicle movement and the travelled distances were analyzed by creating kymographs of axonal segments of transfected neurons imaged for 25 s at the rate of 4 Hz. Continuous vesicle traces without stop or change of direction were counted as single events and measured for their velocity and distance. Although there was a slight tendency for an increased travelling distance of the GFP-Bsn^S2845A^ the statistical analysis did not reveal significant difference between velocities (1.45±0.35 µm/s for GFP-Bsn vs. 1.48±0.28 µm/s for GFP-Bsn^S2845A^; mean±SEM; n = 6 vs. 8 videos; 539 vs. 304 traces respectively, P = 0.8457, unpaired t-test) or travelling distances (4.91±2.06 µm vs. 6.10±2.16 µm; n = 6 vs. 8 videos; 539 vs. 304 traces respectively P = 0.1660, unpaired t-test). The results imply that the disruption of the 14-3-3 interaction site on Bassoon does not significantly influence the transport properties of the Bassoon transporting vesicles. The relative mobility of these vesicles was analyzed by counting the moving and stable GFP-Bassoon and GFP-Bsn^S2845A^ puncta in the videos during a time period of 25 s (100 frames). The relative percentage of immobile puncta of GFP-Bsn did not significantly differ from GFP-Bsn^S2845A^ (92±0.6% vs. 93±0.7 of total puncta; n = 20 vs. 18 videos; P = 0.1020; unpaired t-test). Taken together, the lower recovery rate of GFP-Bsn^S2845A^ cannot be explained by a defect in the supply of Bassoon from the mobile pools to the synapse. We therefore propose that the S2845 mutation in Bassoon leads to a stronger anchoring to the presynaptic cytomatrix (or to a lower rate of dissociation from it), what eventually leads to the observed lower exchange rate.

## Discussion

The integrity of the CAZ is essential for properly controlled release of neurotransmitter from presynaptic terminals [5], [43], [44], [45], [46]. Although this tightly cross-linked protein network is regarded as quite stable it becomes increasingly clear that dynamically regulated modifications of its protein constituents and alterations of its composition play an important role in synaptic plasticity processes [20], [21]. However, a causative link between rapid modification of particular CAZ components and changes in their molecular dynamics has not yet been established. In this study, we describe a novel interaction of the presynaptic scaffold protein Bassoon with the small adaptor protein 14-3-3. We show that the association of 14-3-3 with Bassoon is dependent on a specific phosphorylation of Bassoon at its residue S2845 and identify protein kinases of the RSK family that can mediate this phosphorylation. Finally, we demonstrate that the mutation of the functional 14-3-3 interaction motif of Bassoon leads to a decrease in the dynamic exchange rates of synaptic Bassoon in neurons. Taken together, we provide here an exemplary mechanism of a rapid molecular modification inducing a switch of protein-protein interaction of Bassoon and controlling its dynamic association with the presynaptic cytomatrix.

### pS2845 of Bassoon mediates its interaction with 14-3-3

Protein phosphorylation is a fast and reversible way to modulate protein function and was recognized to induce rearrangements of numerous protein complexes in processes of synaptic plasticity [47], [48], [49]. In recent years, three independent proteomic studies [7], [8], [9] identified 20 to 30 phosphorylation sites on Bassoon constituting it as one of the most highly phosphorylated synaptic proteins. Nevertheless, no functional mechanism mediated by any of the described phosphorylation sites of Bassoon had been established to date. In this study, we identified the phosphorylated S2845 of Bassoon to mediate the interaction of Bassoon with the small adapter protein 14-3-3. We identified the interaction of Bassoon with 14-3-3η in an unbiased Y2H screen. Using interaction studies in vitro, in mammalian cell lines and in primary hippocampal neurons we demonstrated the critical dependence on the intact serine residue 2845 of Bassoon and its phosphorylation for its binding to 14-3-3. Further we showed that the ectopic expression of the interaction motifs e.g. on mitochondria is capable to drive a redistribution of the interaction partner in living cells. Besides the initially found 14-3-3η isoform also isoforms β, γ and ε could interact with Bassoon. This is in agreement with the previously reported highly overlapping target motif preferences of 14-3-3 proteins [30] causing interaction of several 14-3-3-binding partners with multiple 14-3-3 isoforms [50], [51], [52].

### S2845 of Bassoon can be phosphorylated by RSKs

Our in vitro experiments showed that binding of 14-3-3 to Bassoon critically depends on the phosphorylation of Bassoon S2845. Multiple unbiased proteomic studies identified the phsophorylation of Bassoon S2845 [7], [8], [9], which ultimately confirms the physiological occurrence of this modification in vivo. In this study we introduced a newly generated phospho-specific antibody against this residue. This antibody specifically recognized heterologously expressed phosphorylated Bassoon. Unfortunately, we failed to detect the phosphorylated S2845 in mouse or rat brain lysates, probably due to low abundance or transient nature of the phosphoepitope under normal conditions. However, we were able to detect the phosphorylated S2845 in brain lysates supplemented with Mg^2+^/ATP upon incubation at 30°C or in lysates of cells treated with the protein phosphatase inhibitor okadaic acid (data not shown) demonstrating that this epitope can be phosphorylated by endogenous kinases. Interestingly, okadaic acid – induced phosphorylation of S2845 was reverted by pretreatment of cells with the specific RSK family inhibitor BI-D1870 (data not shown), further supporting role of RSKs in the phosphorylation of Bassoon at this residue.

The α-pS2845 Bsn antibody was instrumental for identification of the RSK family kinases to phosphorylate the S2845 of Bassoon in in vitro phosphorylation assays. RSK1 and 3 were about twice as potent compared to RSK2 and 4 in vitro. All RSKs genes are expressed in the nervous system, having restricted but overlapping developmental and regional expression patterns [53] and they have been suggested to share partial functional redundancy [54]. Thus, different RSKs might be involved in the phosphorylation of S2845 of Bassoon depending on developmental stage and brain area. RSK1 is expressed during early embryogenesis, whereas RSK3 becomes the most abundant RSK in the fetal and neonatal stages [54]. RSK3 is therefore the best candidate to phosphorylate S2845 of Bassoon in the juvenile stages. In adult brain, RSK1 is most strongly expressed in cerebellar granular cells, whereas RSK2 and 3 are abundant in forebrain structures. RSK2 was found to be mutated in the Coffin-Lowry syndrome, a disorder characterized by psychomotor and growth retardation [55], which was proposed to be a consequence of selective defect of RSK2 function in hippocampus and cerebellum [54]. Interestingly, there is a possible convergence with the phenotype of Bassoon mutant mice that show altered short-term plasticity in cerebellar mossy fiber to granule cell synapses and in mossy fiber synapses in the hippocampal CA3 region [5], [56].

### Role of the 14-3-3 interaction with Bassoon

14-3-3 proteins frequently function as dimers 1) to induce a conformational change of target protein by interacting with two interaction sites on the same protein and clamping it, 2) to stabilize protein complex formation by bridging two 14-3-3 interaction partners, or 3) to inhibit protein-protein interactions by competing for binding sites [12]. The interaction of Bassoon with 14-3-3 was fully disrupted in vitro and in cellular context by mutation of S2845 suggesting existence of a single 14-3-3 interaction interface on Bassoon. Therefore, we consider the first scenario as unlikely. In our FRAP experiments we observed lowered recovery rates of GFP-Bsn^S2845A^ suggesting that 14-3-3 binding favors dissociation of Bassoon from its presynaptic anchor. It is also possible 14-3-3 associates with free Bassoon and interferes with its association. In either scenario phosphorylation of S2845 and binding of 14-3-3 decreases attachment of Bassoon to the presynaptic CAZ. The complex and tightly interwoven character of this presynaptic protein meshwork is caused by diverse interactions between the single CAZ constituents [1]. Bassoon interacts with CAST/ELKS2 [57], which in turn can interact with Piccolo and RIM [57], [58]. Furthermore, liprin-α also interacts with RIM and CAST [45], [59]. It appears likely that molecular remodeling of such complex protein network requires loosening of intermolecular interactions of its components. Interestingly, Bassoon is not the only 14-3-3 interacting CAZ component. The interaction of 14-3-3 with RIM was suggested to be critical for the induction of presynaptic LTP [60], although this was controversially discussed later and the absence of a phosphorylation site in RIM did not cause an identifiable phenotype in vivo [61], [62] CAST and liprin-α were found to bind 14-3-3 in two independent proteomic screenings for 14-3-3 interaction partners [63], [64] but the function of these interactions was not investigated yet. We suggest, that phosphorylation of CAZ components and their binding by 14-3-3 might support their solubilization by interfering with the intermolecular interactions among them. Supporting this hypothesis, a treatment of neurons with okadaic acid, which increases phosphorylation of serine in the 14-3-3-binding motifs of RIM [60] lead to rapid solubilization and diffusion of cytomatrix proteins Bassoon, CAST and RIM and to a disruption of the synaptic vesicle pool without affecting the postsynaptic scaffolds (SR, unpublished data).

During synaptogenesis membrane-associated Bassoon is transported on Piccolo-Bassoon transport vesicles from the cell bodies to the distal axons to be inserted into nascent synapses [25], [40], [41]. An assembly of a complex protein meshwork at the cytoplasmic surface of transport vesicles could lead to steric hindrance of the transport process. Therefore, a phosphorylation-induced and 14-3-3-assisted masking of the binding sites might be favorable at this stage. Activity-dependent synaptic plasticity goes hand in hand with profound rearrangement of the CAZ [20], [21] but the underlying mechanisms are still unclear. It is an appealing hypothesis that rapid and specific phosphorylation of CAZ components might regulate their binding to each other and induce the molecular remodeling during processes of synaptic plasticity. Indeed, the over-expression of a 14-3-3 dominant negative mutant inhibits the LTP induction in the cerebellum, which was discussed in the connection of its interaction with the CAZ protein RIM [65]. What will be the consequence of a specific interference with the interaction between 14-3-3 and Bassoon in the brain is an exciting question for future studies.
